# Supporting Children’s Social Connection and Well-Being in School-Age Care: Mixed Methods Evaluation of the Connect, Promote, and Protect Program

**DOI:** 10.2196/44928

**Published:** 2023-07-25

**Authors:** Alyssa Clare Milton, Zelalem Mengesha, Kristin Ballesteros, Tom McClean, Saskia Hartog, Lucie Bray-Rudkin, Cathy Ngo, Ian Hickie

**Affiliations:** 1 Central Clinical School, Faculty of Medicine and Health University of Sydney Camperdown Australia; 2 Brain and Mind Centre, Faculty of Medicine and Health University of Sydney Sydney Australia; 3 ARC Centre of Excellence for Children and Families over the Life Course Sydney Australia; 4 Research and Social Policy Unit Uniting North Parramatta Australia; 5 Centre for Health Equity Training University of New South Wales Kensington Australia; 6 Children, Youth and Families Uniting North Parramatta Australia

**Keywords:** participatory design, evaluation, children, school-age care, after-school care, health, well-being, program development, community consultation

## Abstract

**Background:**

School-age care, such as outside school hours care (OSHC), is the fastest-growing childhood education sector in Australia. OSHC provides a unique opportunity to deliver programs to enhance primary school–age children’s social, emotional, physical, and cognitive well-being.

**Objective:**

This study aimed to pilot the co-designed Connect, Promote, and Protect Program (CP3) and conduct formative and process evaluations on how well the CP3 achieved its intended aims, ascertain areas for improvement, and determine how the CP3 model could be better sustained and extended into OSHC settings.

**Methods:**

A naturalistic formative and process evaluation of the CP3 implementation was undertaken at 1 and then 5 OSHC sites. Qualitative and quantitative feedback from stakeholders (eg, children, OSHC educators, volunteers, and families) was collected and incorporated iteratively for program improvement.

**Results:**

The formative and process evaluations demonstrated high program engagement, appropriateness, and acceptability. Co-design with children was viewed as highly acceptable and empowered children to be part of the decision-making in OSHC. Feedback highlighted how the CP3 supported children in the 4 CP3 domains: Build Well-being and Resilience, Broaden Horizons, Inspire and Engage, and Connect Communities. Qualitative reports suggested that children’s well-being and resilience were indirectly supported through the Broaden Horizons, Inspire and Engage, and Connect Communities CP3 principles. Matched-sample 2-tailed *t* tests found that children’s prosocial behaviors increased (mean difference=0.64; *P*=.04; *t*_57_=−2.06, 95% CI −1.36 to −0.02) and peer problems decreased (mean difference=−0.69; *P*=.01; *t*_57_=2.57, 95% CI 0.14-1.13) after participating in the CP3. Program feasibility was high but dependent on additional resources and CP3 coordinator support.

**Conclusions:**

To our knowledge, the CP3 is the first co-designed well-being program developed and evaluated specifically for OSHC services. This early evidence is promising. The CP3 may provide a unique opportunity to respond to the voices of children in OSHC and those that support them through creative and engaging co-designed activities. Our research suggests that CP3 provides OSHC with a framework and high-quality program planning tool that promotes tailored interventions developed based on the unique needs and preferences of those who will use them.

## Introduction

### Background

Approximately, 1 in 5 primary school–age children in Australia are vulnerable to developmental delay, which can affect well-being (social, emotional, and physical), language, and cognitive skills [1]. Furthermore, the second Australian Child and Adolescent Survey of Mental Health and Wellbeing (Young Minds Matter) found that, among primary school students, an estimated 18.2% of boys and 12.4% of girls had experienced a mental health–related disorder in the previous 12 months [2]. To address childhood vulnerability, the Organization for Economic Co-operation and Development [3,4] calls for increasing focus on child well-being programs and optimizing educational environments. A critical strategy includes harnessing existing educational structures and broadening the scope of educational curricula to target children’s health and well-being. Importantly, this includes delivering programs not only during formal school hours but also in school-age care, which encompasses outside school hours care (OSHC), before- and after-school care, vacation care, and leisure-time centers [3].

School-age care, such as OSHC, is the fastest-growing childhood education and care sector in Australia [5]. In 2020, the Productivity Commission reported 5000 OSHC sites supporting 460,000 Australian children. OSHC services offer a secure and supervised environment for primary school–age children before and after school, generally for 2 to 3 hours a day during the school term [6], and offer vacation care during school holidays. In Australia, school-age care services can be provided in schools or community facilities by for-profit and not-for-profit organizations and are regulated by the National Quality Framework and National Quality Standard of the Australian Children’s Education and Care Quality Authority (ACECQA) [7,8]. School-age care services such as OSHC provide an essential service for many families by enabling parents and primary caregivers to achieve a balance between childcare, social responsibilities, and work beyond regular school hours [9]. However, a recent New South Wales (NSW) Department of Education review reported that the standard of well-being–focused initiatives in the OSHC sector needed improvement [5]. The review’s recommendations called for OSHC sites to extend beyond providing “convenient care” [5] and, instead, be a place where children’s well-being is actively supported. Indeed, OSHC offers a unique opportunity to implement prevention and early intervention programs designed to multidimensionally enhance children’s health and well-being [10].

As such, there has been increased attention from researchers, educators, the government, and the broader community toward how specific well-being–focused programs delivered during OSHC could be better used to support children’s learning and growth. Such programs need to be researched, and the OSHC community should be an active research partner [5], through co-design. This is important as OSHC services differ considerably in geography, community context, educator expertise, and the number and characteristics of the children who attend. Programs that are suitable for one OSHC service may not be feasible or appropriate for another [6].

The only known well-being–focused OSHC program that has been developed in Australia through the use of co-design is the Connect, Promote, and Protect Program (CP3) [6]. Co-design, also known as participatory design, places stakeholders at the center of the design process [11,12]. It enables a paradigm shift toward collaborative bottom-up engagement whereby stakeholders (eg, OSHC children, educators and volunteers, and parents or guardians) jointly explore and create solutions for program design and service delivery [6]. Essentially, participatory design allows programs to be co-designed with the people who use them.

**Figure 1:**
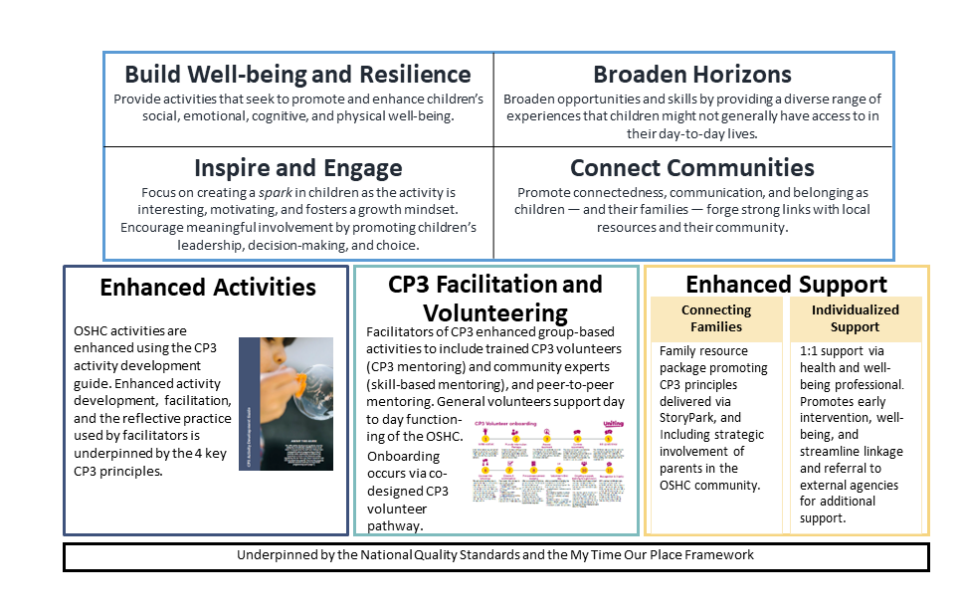
Connect, Promote, and Protect Program (CP3) model. OSHC: outside school hours care.

CP3 is a structured method for co-designed OSHC activity program development and delivery. It provides opportunities for social connection, child leadership, and engagement and delivers activities that broaden children’s experiences, opportunities, and well-being. As shown in the CP3 model (Figure 1) and discussed in previous co-design research [6], the CP3 has four guiding programming principles: (1) Build Well-being and Resilience, (2) Broaden Horizons, (3) Inspire and Engage, and (4) Connect Communities. CP3 is the only cited example of co-designed research in the OSHC space in the Department of Education review of OSHC services [5].

### Objectives

This study reports on the formative and process evaluation of the CP3 model through a partnership between the University of Sydney’s Brain and Mind Centre (an Australian multidisciplinary research institute focusing on conditions that affect child development, youth mental health, and brain aging) and Uniting (formally Uniting Care NSW.ACT, a large provider of children’s services, including before- and after-school care, vacation care, occasional care, long-day care, and preschool in NSW, Australia). The main objective of this study was to pilot the co-designed CP3 in real-world school-age care services to conduct a formative and subsequent process evaluation. The purpose of the evaluation was to establish how well the CP3 achieved its intended aims, ascertain areas for improvement, and determine how the CP3 model could be better sustained and extended to OSHC settings. Therefore, the research questions were as follows: (1) How well did the CP3 achieve its intended aims in terms of engagement, appropriateness, acceptability, feasibility, and preliminary effectiveness? and (2) What were the barriers, facilitators, and areas for improvement regarding CP3 implementation to inform future program delivery?

## Methods

### Ethics Approval and Compliance With Ethical Standards

This study was approved by the University of Sydney Human Research Ethics Committee (protocol 2018/832). All procedures were performed in accordance with the with the 1964 Helsinki Declaration and its later amendments or comparable ethical standards. All data, including the images and figures in this publication, are presented in nonidentifiable formats.

### Study Design

This study was a naturalistic formative evaluation and subsequent process evaluation of the CP3 model in OSHC services. The CP3 model was developed with local stakeholders in 2017 using participatory design and is reported elsewhere [6]. A mixed methods evaluation was used in this study, including the collection, analysis, and interpretation of quantitative and qualitative data [13]. The overarching CP3 research is based on the Medical Research Council guidelines for developing complex interventions [14], which use an iterative research design cycle of development, feasibility, evaluation, and implementation.

### Participants and Setting

In both the formative and process evaluations, participants comprised three stakeholder groups: (1) children attending OSHC, (2) OSHC volunteers (ie, CP3 principle mentors and CP3 skilled mentors, defined in the *CP3 Roles* section) or educators (including managers), and (3) parents or guardians of children attending OSHC. The inclusion criteria were (1) being identified as belonging to one of the stakeholder groups, (2) ability to participate in English, and (3) the provision of written informed consent to participate. For a child to participate in any evaluation, both parental or guardian and child written consent were obtained. For the formative evaluation, participants were both children and adults recruited from an OSHC site in the Illawarra region of NSW, Australia, between July 2019 and June 2020. For the subsequent process evaluation, participants were both children and adults recruited from 5 OSHC sites in the Sydney region of NSW, Australia. The CP3 was implemented at these sites in 2020 (term 4) and 2021 (term 2) over a 10-week period.

### CP3 Roles

There are multiple CP3 roles andCP3 personnel who support CP3 delivery, and they will be discussed throughout the reporting of the evaluation findings. This includes the roles outlined in Textbox 1.

**The roles in the Connect, Promote, and Protect Program (CP3).**

**CP3 coordinator**

- This is the overarching coordinator of the CP3, who supports sites implementing the CP3 through resourcing, training, activity planning, delivery, and evaluation.

**CP3 site champion**

- This is the nominated educator who is responsible at a site level for supporting CP3 delivery.

**CP3 skilled mentors**

- Skilled mentoring complements the range of activities that can be provided as part of the CP3. These champions are mentors with specialized skills that can facilitate activities in their areas of expertise—whether it be movie making, martial arts, or community advocacy. Depending on their availability, skilled mentors can help facilitate one-off sessional activities or a full CP3 activity program or they may simply offer outside school hours care (OSHC) sites the use of specialized resources.

**CP3 principle mentors**

- These mentors are trained in and have an in-depth understanding of the
  CP3 principles
  (ie, Build Well-being and Resilience, Broaden Horizons, Inspire and Engage, and Connect Communities). Their role is to support the CP3 activities each week to ensure that the CP3 principles are being delivered in each session.

**CP3 peer champions**

- These are children attending OSHC sites who are particularly interested in the CP3. These peer champions can play a variety of roles depending on the OSHC. For example, they might lead CP3 announcements in the OSHC community meetings or buddy up with other children who might need additional support during CP3 workshops or CP3 activities.

### Recruitment and Informed Consent

Electronic and paper-based advertising materials were used to notify potential participants (as well as students’ parents or guardians) of the study. Recruitment was passive so that participants (or their parents or guardians) initially volunteered by contacting researchers to participate, or they could directly take part by completing the survey (paper-based or web-based depending on participant preference). After parental consent was obtained, children went through a consent and a subsequent assent process immediately before the activity. All individuals completed an informed consent process before participating in this research. All participants were reassured of the voluntary nature of participation and that they could stop at any time. Participants did not receive any compensation or reward for taking part in the research; however, all workshops were catered.

### Outcomes

For the formative evaluation, a survey collected details such as gender, age, postcode, language spoken at home (children only), year at school (children only), relationship with OSHC site (adults only), satisfaction with the OSHC service, social connectedness (measured using the 1-item Inclusion of Community in Self scale [15]), and quality of life (measured using the Personal Wellbeing Index [16,17]). For the process evaluation, the primary outcome was measured using changes in Strengths and Difficulties Questionnaire (SDQ) [18] scores from baseline to the end of the CP3. The SDQ was already used by OSHC educators for routine monitoring and, thus, aligned with naturalistic service delivery and minimized additional administrative burden. The SDQ is a 25-item behavioral screening questionnaire with 5 scales (emotional symptoms, conduct problems, hyperactivity or inattention, peer relationship problems, and prosocial behaviors). The SDQ has sound psychometric properties in Australian samples of children (aged 4-9 years) [19]. Demographic details such as child gender and age were also available from OSHC routine data monitoring. For both stages of the evaluation, qualitative data related to CP3 acceptability and feasibility were gathered through surveys, participatory design workshops, and routine data outcome monitoring interviews.

### Data Analysis

#### Qualitative

Qualitative data sources and artifacts from participatory design workshops and interviews included detailed notes from workshops by the research team, deidentified transcription notes from routine data outcome monitoring, and notes written by participants on handouts and worksheets. Qualitative data were analyzed using a six-step qualitative thematic analysis [20]: (1) data familiarization; (2) generating initial codes; (3) searching for themes and subthemes; (4) reviewing themes; (5) refining, defining, and naming themes; and (6) report writing. This stepwise process provides a flexible and accessible way of analyzing qualitative data and enables iterative exploration of patterns and relationships between different themes while ensuring research rigor. All qualitative data sources from the workshops and interviews were reviewed by 3 researchers (KB, ZM, and AM), who noted relevant points and key concepts across all participants to develop an initial coding framework. The pattern of themes generated from the data was mapped back to the CP3 principles (Build Well-being and Resilience, Broaden Horizons, Inspire and Engage, and Connect Communities), program satisfaction, program challenges, and educator and volunteer outcomes. This became the framework matrix used for coding the data [21]. Notes were then coded in the NVivo software (version 11; QSR International) [22] using this framework by 2 researchers per transcript (SH and LBR), and any discrepancies were discussed with a third researcher (AM). Coding followed an iterative process of reading, coding, and discussing the pattern and content of the coded data.

#### Quantitative

Owing to the small number of participants in the formative evaluation, statistical analysis of the quantitative data generated from the child and adult evaluation surveys was descriptive only. For the process evaluation, SDQ scores from routine data outcome monitoring were used. SDQ scores from scales 1 to 4 were added to obtain a total difficulties score. The SDQ recommends a four-fold classification: (1) close to average, (2) slightly raised or lowered, (3) high or low, and (4) very high or very low. Analysis of SDQ data was performed using SPSS (version 28; IBM Corp). Participant data were matched across baseline and follow-up data. After a 1-sample *t* test (2-tailed) was performed to ensure that there was no difference between the matched and full sample in baseline emotional and behavioral difficulties SDQ scores, a matched-sample *t* test was performed to analyze the mean difference (MD) between baseline and follow-up scores. The post hoc calculation for the matched sample indicated that we could detect a small to medium effect size (0.38) at 80% power (2-tailed; Cronbach α=.05) with the achieved sample (n=58).

## Results

### Formative Evaluation

#### Implementation Phases

The CP3 model and implementation process (stages 1 to 3) were tested during the formative evaluation.

##### Stage 1: Consult and Create

During the first school term, CP3 implementation commenced with initial community consultation to provide information about the CP3 and obtain an early understanding of the needs and wants of the OSHC community. This was followed by CP3 training and participatory design workshops with educators or volunteers (n=6 in 1 workshop) and then 90-minute participatory design workshops with a proportion of the OSHC children (n=16 in 3 workshops) who had parental consent to co-design the CP3 activities for planned delivery. These were facilitated by a psychologist with support from the CP3 site champion (an OSHC educator) and the CP3 coordinator, who took detailed session notes. The main purpose of the child workshops was to engage children in decision-making and planning for the upcoming CP3 enhanced activities. This consisted of four stages: (1) discovery: exploring the children’s activity interests; (2) evaluation: understanding children’s preferences for the different enhanced activity ideas that had been cocreated in previous workshops with children and educators; (3) mapping: obtaining further information on how children think the activities are linked to the 4 CP3 principles; and (4) prototyping: encouraging children to cocreate their own CP3 activity. The outcome of the *consult and create* phase was 3 activities focusing on promoting physical activity, creative pursuits, and skill development, which were further enhanced by educators and children during the participatory design sessions to align with the CP3 principles. An example activity is provided in Textbox 2.

**Example activity.**

Example

- A popular activity co-designed in the *consult and create* stage that was highly acceptable to children was Woodwork Café. Regarding Connect, Promote, and Protect Program principles, children envisioned that the activity would make them feel connected to their families as it was an activity they could do with them; children could give (or sell) the things they built to others in the community (*Connect Communities*); the activity could be a cognitive challenge for their brain and fun and, therefore, make them happy (* Build Well-being and Resilience*); and the activity would be exciting and fun to do (*Inspire and Engage*) and allow them to build things they had not made before and use their imaginations (*Broaden Horizons*).

##### Stage 2: Test and Refine

Ideas generated in the *consult and create* phase were actively applied via a taste-tester program. All the children attending the OSHC site were able to participate in this program during the second school term. Multiple co-designed activities generated in stage 1 (Woodwork Café, Movie Maker, Get Active, and Art Space; Textbox 3) were tested, and feedback from children, volunteers, educators, and families was collected.

**Connect, Promote, and Protect Program (CP3) implementation stages.**

**Figure 2:**
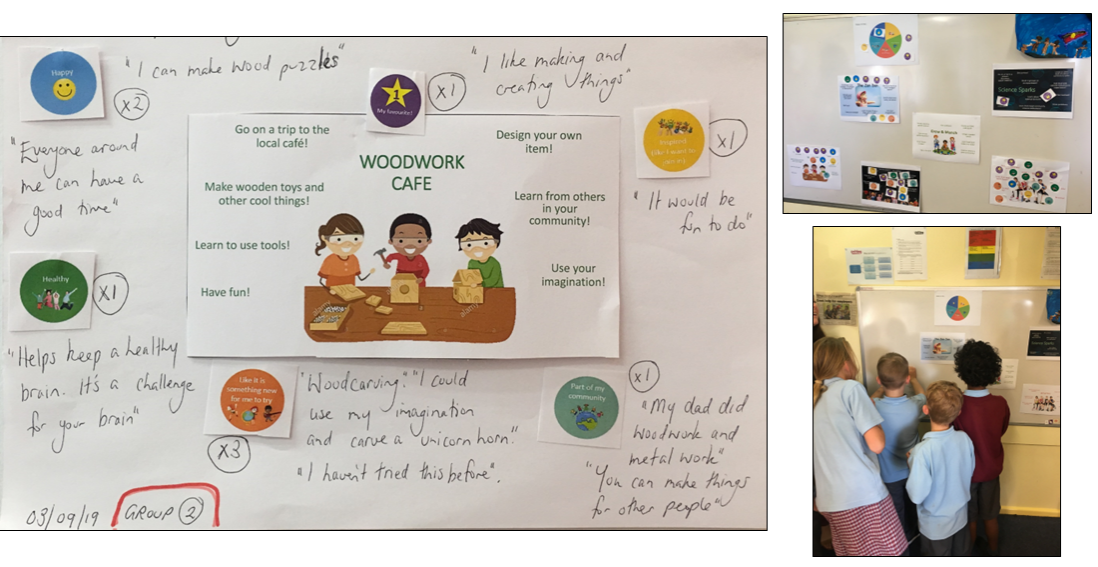
Participatory design artefact—Woodwork Café.

**Stage 1: activities co-designed by children and educators—term 1 (Figure 2)**

- Woodwork Café: this was a program focused on developing woodworking skills.
- Movie Maker: this was a program focused on script writing, performance, and film production.
- Get Active: this was a program focused on physical activity.
- Art Space: this was a program focused on well-being and creative expression through art.
- Science Sparks: this was a program focused on engaging Science, technology, engineering, mathematics (STEM) activities.
- The Zen Den: this was a program focused on mindfulness and nature.
- Farm to Fork: this was a program focused on gardening and cooking skills.

**Stage 2: co-designed activities selected and trialed in taste-tester sessions at the outside school hours care (OSHC) site—term 2**

- Woodwork Café: volunteers (trained as CP3 principle mentors) supported children to learn to use tools to build 2 go-carts for the OSHC. The children then raced the go-carts in teams at a community event. The program built their planning and communication skills, fine and gross motor skills, and teamwork and community connection.
- Movie Maker: the children taste-tested drama sessions with a volunteer drama teacher (a CP3 skilled mentor) to build their confidence in acting and speaking in public and community links with the local drama school.
- Get Active: the children taste-tested lawn bowls with local volunteers (trained as CP3 principle mentors) at the local community club, building community connection, intergenerational bonds, teamwork, and gross and fine motor skills.
- Art Space: a young local person and artist (CP3 skilled mentor) volunteered to run an art class with the children, and peer-to-peer mentoring also took place with older children (CP3 peer mentors) supporting younger ones. Children worked collaboratively to create an art project that was based on their own choosing for exhibition at the OSHC.

**Stage 3: co-designed activities selected and implemented as a full program—term 3**

- Woodwork Café was selected by children and educators to continue as a larger program in the final CP3 term. Children, educators, parents, and volunteers collaboratively contributed and co-designed what the program would look like and how it met the CP3 principles. In brief, for this full-term CP3 activity, volunteers (trained as CP3 principle mentors) from the local community supported children in building their own chicken coop at the OSHC site. A volunteer brought a chicken for an OSHC site visit so children could interact with and learn about chickens before baby chickens arrived on-site. Furthermore, an OSHC family provided the CP3 with 2 baby chickens and an incubator to hatch the eggs to live in the coop. Children could engage on the web as well as on-site to see the chickens hatching. The process was documented from beginning to end with updates and photos, which could be shared in paper-based and web-based formats with the OSHC community, enhancing children and their family’s engagement and excitement. The co-designed program connected children with their community, broadened their horizons through skill development, and fostered child leadership and well-being. For example:
  Cognitive well-being: through active collaborative planning—the children actively researched how to care for chickens and the best options for chicken coops.
  Physical well-being: fine and gross motor skills were developed through learning to use tools.
  Social well-being: children worked as a team and communicated with each other about the planning and the building of the chicken coop, and intergenerational connections were made with the community volunteers and another local preschool (the children shared resources and ideas about chickens and coops with younger children).
  Emotional well-being: information about the well-being benefits of animals (eg, companionship, soothing capacity, and happiness promotion) was discussed with children and families verbally and via a web-based communication platform. Well-being resources were shared with families related to strategies for managing anxiety and boosting resilience.

##### Stage 3: Implement and Evaluate

This last phase involved the implementation and evaluation of a full-length CP3 activity program after incorporating feedback from the *test and refine* phase delivered over a full school term with the full cohort of children attending the OSHC site. In stage 3, the children and educators collaboratively selected which co-designed program they would like to undertake from the taste testers, and they subsequently participated in the third consecutive school term. Examples of the co-design and implementation of the activities over all 3 stages are presented in brief in Textbox 3.

#### CP3 Formative Evaluation Feedback

*Engagement* with the CP3 formative evaluation pilot program was high, with educators reporting that most children at the pilot site engaged in the program, including children with additional support needs. Participation rates were an average of 10 to 15 children per session throughout CP3 implementation.

The *appropriateness and acceptability* of the CP3 formative evaluation pilot program were rated as very high based on child, educator, and CP3 volunteer (ie, CP3 principle mentors and CP3 skilled mentors) feedback (demographics are presented in Multimedia Appendix 1). Specifically, the average endorsement by children on CP3 target areas (feeling happy and talking about feelings, staff listening, having fun and making their own choices, and learning and trying new things) across all time points was high—ranging from 84% (16/19) of the children agreeing that they could make their own decisions in OSHC to 100% (22/22) agreeing that the CP3 at the OSHC site was fun.

Table 1 summarizes adult participant endorsement rates (agree to strongly agree) with the CP3 principles demonstrated over the program timeline.

To assess CP3 *feasibility*, workplace-related satisfaction questions were presented to educators and volunteers at time point 3 (Multimedia Appendix 2), and a focus group was run. Volunteers were satisfied or very satisfied with all the items. Educators were satisfied to very satisfied with most items, with paperwork and being part of the decision-making at the OSHC site receiving the lowest satisfaction scores (3/5, 60% of the educators being satisfied or very satisfied). The workshop yielded highly positive feedback on the CP3. Ideas were collated from this to form a CP3 implementation guide for future implementation and the process evaluation at additional OSHC services.

**Table 1:** Adult participant endorsement rates (agree to strongly agree) with aggregated Connect, Promote, and Protect Program (CP3) principle evaluation items.

CP3 principle	Items	Baseline (before CP3 engagement; n=5), item endorsement rate, n/N (%)	Time point 2 (after CP3 taste-tester activity; n=9), item endorsement rate, n/N (%)	Time point 3 (end of CP3; n=18), item endorsement rate, n/N (%)
Build Well-being and Resilience	Social well-being Emotional well-being Cognitive well-being Physical well-being Resilience	20/50 (40)	38/42 (90)	87/90 (97)
Broaden Horizons	Broaden children’s skills Diverse range of experiences	4/10 (40)	18/18 (100)	35/36 (97)
Inspire and Engage	Interesting Motivating Believing that their skills and talents can grow Part of the decision-making Leadership skills	13/25 (52)	39/44 (89)	83/90 (92)
Connect Communities	Strong links with local resources Strong links with local community Connectedness and belonging of children and their families Communication with children and their families	8/20 (40)	29/35 (85)	65/71 (92)

### Process Evaluation

The number of returned SDQs completed by educators for each participating OSHC site is presented in Multimedia Appendix 3. Site 5’s follow-up data collection was affected considerably by the COVID-19 lockdown (7/32, 22% completion rate), and site 1 did not collect SDQ data because of workforce capacity issues. The SDQ scores for all participants at all sites are presented in Multimedia Appendix 4.

Of the 88 students who had SDQ data collected at follow-up, 58 (66%) could be matched. A total of 81% (47/58) of these students were female and had a mean age of 7.9 (SD 1.9) years. The baseline total emotional and behavioral difficulties SDQ scores for the matched-sample subset did not differ significantly from those of the full baseline sample in the 1-sample *t* test (8.95 vs 9.08; *P*=.28; *t*_89_=1.08, 95% CI −0.09 to 0.32). A matched-sample *t* test analysis of matched students found that prosocial behaviors significantly increased (MD=0.64; *P*=.04; *t*_57_=−2.06, 95% CI −1.36 to −0.02) and peer problems significantly decreased (MD=−0.69; *P*=.01; *t*_57_=2.57, 95% CI 0.14-1.13) after participating in the CP3 (Table 2). All other changes in SDQ items (emotional symptoms, conduct problems, and hyperactivity) were not statistically significant (all *P*>.05; Table 2).

**Table 2:** Changes in Strengths and Difficulties Questionnaire items for the matched sample (n=58).

	Mean difference (SD; 95% CI)	*t* test (*df*)	*P* value (2-tailed)
Emotional symptoms	−0.10 (1.71; −0.55 to 0.35)	−0.46 (57)	.65
Conduct problems	0.22 (1.95; −0.29 to 0.74)	0.87 (57)	.39
Hyperactivity	0.38 (2.43; −0.26 to 1.02)	1.19 (57)	.24
Peer problems	0.64 (1.89; 0.14 to 1.13)	2.57 (57)	.01
Prosocial behaviors	−0.69 (2.55; −1.36 to −0.02)	−2.06 (57)	.04

### Qualitative Evaluation

#### Overview

In total, 24 adults provided qualitative evaluation feedback (n=3, 12% parents or guardians; n=2, 8% volunteers; n=15, 62% educators; and n=4, 17% coordinators or managers) in interviews and focus groups. In total, 2 child workshops with 11 children also took place after the CP3 process evaluation. The pattern of themes from the data was mapped back to the original CP3 principles and, therefore, was used in a framework analysis approach alongside reporting program satisfaction, program barriers, and impact on educators and volunteers.

#### Program Satisfaction

##### Child Satisfaction

Child satisfaction with the CP3 was very high, with children describing it as a “more fun than normal OSHC day” (child; workshop 2). A number of children recognized that other children also enjoyed the CP3—“when you do this activity everyone is running to do it” (workshop 1)—and expressed a desire for more CP3 activities:

I wish I could do CP3 more often.

Workshop 2

...make OSHC better by having more stuff to do like this.

Workshop 1

Adult participants echoed that children thoroughly enjoyed and looked forward to OSHC when the CP3 was on, particularly because of differentiation from normal OSHC days:

Oh, she loves it very much. She really looks forward to going to it...she enjoys the different activities and she really likes [educator name], and the educators, and she really enjoys spending time with them.

Parent; P10

##### Family Satisfaction

The CP3 was seen as strengthening the relationship between families and OSHC, enabling families to be more involved in decision-making, and increasing awareness of their children’s talents and capabilities:

It leads to happy parents. Parents are happy to see the different, the new, the challenging activities. Children learning a different sport or a different skill or giving back to the community, which, that feedback gets fed through to the provider, to the service.

OSHC manager; P7

##### Volunteer and Mentor Satisfaction

CP3 volunteers and mentors relayed that they benefited personally, felt proud of what they had achieved, developed new skills, and felt more connected to their community. Volunteering had a ripple effect in the community, where what volunteers taught children was then passed on to others, such as family and friends:

For me as a businessman, it was nice to just sort of be out and about in a different world. It broke up the daily activity. I felt like I was making a difference, which is something is important to me. Anyway, obviously I understand the kids probably don’t get this access. Because that’s why they’re doing after school care, I suppose. They’re not getting the sporting opportunity. So they’re not missing out...at times, like,...giving back in some way, at some point in my life, this was a good engagement for me to see where I’m at as a person, I suppose to start doing that, give back process.

CP3 volunteer; P24

##### Educator Satisfaction

Educators and OSHC coordinators described the CP3 as a highly positive experience as the CP3 was inclusive, unique, innovative, and community-minded. The CP3 supported best-practice program planning and OSHC community engagement and encouraged collaboration and teamwork. The CP3 provided the OSHC with additional resources, equipment, and personnel, which was highly valued. The CP3 principles were reflected in the outcomes for educators personally, which supported their workplace well-being and increased their levels of job satisfaction:

I just think it was a great experience...Yeah. I enjoyed it, and I know the kids enjoyed it, so. No, I don’t think there’s anything much you have to change at all.

Educator; P11

#### Child-Focused CP3 Outcomes

##### Build Well-being and Resilience

The CP3 was described as supporting and building children’s resilience and well-being:

CP3 has been good for the children’s social, emotional, physical wellbeing. They talk a lot about CP3. They enjoy doing it with their friends.

Educator; P1

The CP3 also enhanced well-being indirectly during activities so that children were building skills to support their well-being in a natural and authentic way, including those with underlying challenges or vulnerabilities:

...the kids...struggle with...their emotional regulation...initiating play and interacting with each other is really challenging...Robotics and coding program, they were all initiating in play together, sharing their experience...but also working together as a group and learning through play how to connect with each other through their interests.

OSHC coordinator; P21

Gaps in regular OSHC delivery were highlighted, and the need to scale programs such as the CP3 was suggested:

...there is a definite need and gap in the mental health space for children...I’ve seen it in children as young as five, trying to end their lives...a program like CP3 provides connection, support, acceptance, all these things, the sense of belonging that these children clearly aren’t feeling, and at the age of five that’s heart-breaking. And so, I think CP3...captures those children, that everyone else is missing. The schooling system doesn’t work for them. For some reason, home life isn’t what they want it to be or need it to be. And there’s not a lot we can do about that, but we can create a space in an OSHC through CP3 that supports...and engages those children. It gives them a sense of belonging and so that they know that they matter.

OSHC manager; P7

Most child feedback did not identify well-being directly but, rather, indirectly from other CP3 principles related to learning new things, social connection, helping others, and having fun.

##### Broaden Horizons

One of the most consistent themes was that the CP3 broadened the children’s experiences by creating opportunities to learn new skills or expand their knowledge. The CP3 provided a vast range of new experiences as children were “being exposed to things that just generally wouldn’t be exposed to” (OSHC manager; P7) and facilitated the engagement of children who did not usually participate in OSHC:

The children are excited...“Hey, we’re doing this next week.” It’s supported children in a different sense, where we’re seeing children showing us more of their skills...children who don’t normally participate in activities...we’re actually seeing them participate...

OSHC coordinator; P2

The exposure to new activities fostered future hobbies and interests. Child– and community–co-designed CP3 activities ranged from European handball, cooking, Diwali (festival of lights) celebrations, dancing, soccer, build a bear, woodworking, coding and robotics, crafts, gardening, knitting, and visiting a farm. As activities were co-designed by each OSHC community to uniquely meet their own identified needs and goals, “no two services have been the same” (OSHC manager; P7).

Children commented that “I like it because it’s different” (workshop 1), “It’s different to normal OSHC” (workshop 1), “It means we do new things” (workshop 1), and it “helped me learn how to make things” (workshop 2).

##### Inspire and Engage

The CP3 principle Inspire and Engage aims to create a “spark” in children with interesting activities that motivate and foster growth mindsets. Meaningful involvement in the CP3 is encouraged by promoting children’s leadership, decision-making, and choice:

Child engagement has been fantastic. The children have been so excited to tell us what they want to do at their OSHC. Educators are learning from that too...they say, “Oh, I didn’t know that they were interested in that.” So child engagement and having the child’s voice heard has been very successful.

CP3 coordinator; P4

Each OSHC site varied in how they engaged children in the CP3 decision-making process. Child-led decision-making processes included “using a voting system which kept activities relevant and maintained children excitement” (educators; P13 and P6), “asking the students what they want to do, [and then] incorporating that into [their] program” (educator; P3), using a book in which children could “write down their ideas,” and conducting a survey to decide what activities to do (educator; P8).

An educator highlighted the following:

It was an excellent way of looking at things, seeing what they would like to do in a different way than just asking them, “what’s your interests?”

P14

This made the activities more personally meaningful to the children and resulted in high levels of child engagement and enthusiasm for the CP3. An educator noted that the children selecting the activity and it subsequently being implemented “was a meaningful experience for them because they felt heard” (P13).

Children felt empowered to communicate their opinions:

...we’ve seen children now taking ownership of setting out programs that they want to participate in, really speaking up about what they wanted to do. For example, saying “this is how I feel, I’m not having a turn, I would like more of a turn, or I really want to do this”...We’ve really seen that change in dynamic of that communication now, what I want to do this in taking the ownership of, “Hey, this is my OSHC. This is the program that I want to do.”

Educator; P17

Children mostly reflected the Inspire and Engage theme through their enthusiasm for the program, describing it as “*...*so much fun. Normal OSHC is boring, and school is really boring but CP3 made OSHC a fun thing*.*”

##### Connect Communities

Connect Communities was the most prominent theme identified in the interviews. Participants said that the CP3 helped build children’s relationships and improved their sense of belonging and the way they socialized:

They were all initiating in play together, working together as a group and learning through play how to connect with each other through their interests.

Educator; P18

When they come here to OSHC, it’s all together. We’re all sitting down, we’re all socializing. I think it just brings us all together...and there’s a community spirit.

Educator; P16

This was especially important for children who struggled with emotional regulation and initiating play with others. Multiple participants highlighted that, instead of solitary or small-group play, CP3 activities encouraged purposeful mingling of larger groups:

...this year I can see a change in that child, they are playing in a group now, rather than solitary play.

Educator; P8

Furthermore, the program “provides connection, support, acceptance, all these things, the sense of belonging” (educator; P18).

Children actively developed their interpersonal, collaboration, and teamwork skills by navigating ways to work together and be respectful of each other’s needs and ideas in an inclusive space:

It’s about the safe space, knowing the kids, doing the activities together, finding your own wavelength...everyone working together and sharing their views and just talking to each other and talking your ideas through. It’s just self-confidence...you might not have the right solution, or the right idea, but it’s okay. And being patient to hear each one out as well.

Parent; P19

During CP3 activities, peer-to-peer learning and support occurred organically, whereby children with different skill levels supported each other:

I think the other big part would be the peer-to-peer learning...by the children. I’m thinking of the soccer activity. There were some children who are obviously very skilled in soccer and some that weren’t and the children who were skilled supporting those that weren’t.

CP3 coordinator; P4

At the community level, participants emphasized that the CP3 benefited children by building their relationships with educators through enhanced communication skills, which in turn improved the educator-student relationship (educator; P20).

The CP3 also helped link children to their local community—examples included a partnered senior school or local sporting group. It was viewed as extremely important in supporting a child’s well-being, encouraging a sense of belonging (CP3 coordinator; P4), forging new and important relationships for the future, and being a source of inspiration for the children (educators; P5, P15, and P16). This connection to the community could also make children more aware of their world, have a greater understanding of their community, and feel more like active and connected citizens (OSHC coordinator; P21). Furthermore, the CP3 created an opportunity for children to build a trusting relationship with an adult outside of their immediate family:

It’s another adult in the children’s eyes that they can trust and go to. And if they ever need help, it’s having that relationship there. Which you don’t get, unless you put effort and time into it, which CP3 encourages, in again, that fun setting.

OSHC manager; P7

Children commented on how they enjoyed involving their family and friends:

I like baking and love chocolate, so the cooking was my favourite and my family loved it too. It was more exciting and more fun because I was doing it with my sister and families and friends.

Workshop 2

Helping others in the wider community was also reflected, with a child commenting that “we will help charity” by knitting and “donating [beanies] to other people who need them” (workshop 1) and another child stating that they enjoyed the CP3 as they made “a house for animals affected by the bush fire” (workshop 2)*.*

#### Educator and Volunteer Outcomes

The CP3 was described as a “professional development opportunity” (OSHC manager; P7) that could enhance their engagement and interaction skills with children and make them actively consider child well-being in their practice. A coordinator (P4) reported that educators “definitely gain a lot more depth and understanding on what they [the children] want, need, what makes them tick and how we as a service can best support them*.*” Many comments from service providers indicated that educators connected with children in new and improved ways:

I really connected in a different way or for the first time.

Educator; P6

Another educator (P20) highlighted that the CP3 provided “such a joyful experience” as it enabled educators “to see children in a different way, being able to see them become stronger individuals, learning, taking that opportunity, being motivated*.*” Educators had a greater desire to pay attention to, understand, and act upon children’s wants and needs, which increased their connection with the children.

Job satisfaction and well-being were positively reported:

There’s also been an increased happiness with the educators when they’re participating in the program.

Educator; P22

A CP3 activity that celebrated the Diwali festival of lights resulted in 2 educators feeling proud to share their culture with the children.

Educators also described that CP3 volunteer mentors were “feel[ing] proud” that they “[had come] here as a volunteer and they [taught] something new to the children.” This volunteering had a ripple effect on the local community as they came in to teach something that resulted in “children are teaching their families and cousins and siblings” (educator; P23). Although a volunteer CP3 mentor reported that the CP3 had been a challenging but highly enjoyable experience, helping him “grow further understanding different of things in life with regards to communities and all that sort of stuff,” he emphasized that, through the CP3, “I felt like I was making a difference, which is something important to me” (P24).

#### Challenges to CP3 Implementation

A major challenge to CP3 implementation highlighted by educators was related to the administration of CP3 activities, potentially causing heavier workloads:

...it is a lot of work for us educators. On top of what we already have to do.

Educator; P1

This included engaging in the research (eg, completing SDQ assessments) and sourcing volunteers. Although some OSHC sites delivering the CP3 reported that finding volunteers was an easy process, 20% of the educators advised that sourcing appropriate volunteers from the community was a major barrier. A coordinator described that “the most challenging thing was trying to find volunteers to come in and do activities free of charge” and “it was a challenge to arrange a time that suited both people, especially if it was a teacher or a parent because parents send their kids to OSHC because they work...So not all people are willing to come after they’ve worked to come to OSHC to participate” (P21). Participants highlighted the critical role of a person dedicated to supporting the implementation of the CP3 (the CP3 coordinator) and that this role needed to continue to be embedded in the program.

Some participants reported that educators who were not directly involved in CP3 delivery struggled to explain the CP3 to parents and guardians:

Yeah, how is it valuable, and then that can help them explain it to parents and carers as well because they were struggling to explain, I guess, how the program was going to be...[the CP3 Coordinator] explained it, but they were struggling to deliver that to parents and carers.

CP3 coordinator; P21

Family engagement varied across the participating OSHC sites; some reported excellent engagement, whereas others identified challenges. This was attributed to parents being busy or at work, hence their use of OSHC in the first place. In total, 13% of the educators highlighted that more families at their center wished to be involved, but COVID-19 restrictions prevented engagement. Furthermore, the CP3 coordinator suggested that it may have been a result of “educators not understanding why, how we’re doing this, and prioritizing time” (P4), so further educator training may assist in addressing this.

The level of engagement of the OSHC coordinator was another factor that influenced the success of CP3 implementation:

...the coordinator seems to be the gateway to the inspiration of CP3 within the team...When I was working alongside an engaged, motivated coordinator, it was really, really easy to implement CP3. They put the time and the effort in...Whereas if the coordinator was a bit more distant, a bit more challenging to communicate with, it was more challenging definitely to do...The sparkle wasn’t there. The magic was lost a little.

CP3 coordinator; P4

One of the most cited challenges to CP3 implementation and engagement with the program related to external factors that were beyond OSHC control. This was identified as an issue by many participants and included the COVID-19 pandemic, regional natural disasters, and school protocols in response to such events:

I think out of [OSHC site], we had the fires, then we had poor air quality, then there was flooding. We’ve also had COVID-19...we had some volunteers from the local church congregation who were in their senior years and we weren’t able to re-engage those volunteers as a result of COVID. We sourced volunteers from within the immediate community. So it was educators who might have had a family member, for example. And at [another OSHC site], we had an educator who had a family member who was a soccer coach, so we engaged that person. They were part of the existing community, so we weren’t actually introducing anybody from outside of the immediate community to the space. Schools were quite strict with who came onto the grounds during COVID-19, so that impacted community connections again.

CP3 coordinator; P4

Some OSHC sites were agile and used creative engagement strategies; for example, in response to COVID-19 restrictions, an educator (P22) described purposely setting up the CP3 activities in front of the parent sign-in area so that parents could still engage in some capacity. She also ensured that parents were able to see photos of the activities “so, as much as families couldn’t come in and be involved, they were still involved in a different way.” Other educators (P11 and P12) sought volunteers through family connections and teachers from a linked school.

## Discussion

### Principal Findings

To our knowledge, the CP3 is the first co-designed social connection and well-being program model for primary school–age children (aged 5 to 12 years) specifically for OSHC settings in Australia. Our study used a 2-stage mixed methods formative and process evaluation to assess the acceptability and feasibility of the CP3. These evaluation stages of research are crucial as research suggests that many mentor-style programs are pursued without any supporting evidence from reliable or valid process or outcome evaluations [23,24].

Our evaluation highlights the high level of satisfaction and engagement with the CP3 and demonstrates promising preliminary findings in terms of the positive impact of the CP3 principles (ie, *Build Well-being* and *Resilience*, *Broaden Horizons*, *Inspire and Engage*, and *Connect Communities*). The process evaluation reported significant positive impacts of the CP3 on prosocial behaviors and reducing peer-related problems in children, as measured using the SDQ. These are important findings, especially as the research was conducted from 2019 to 2020 through unprecedented events such as fires, floods, and the COVID-19 pandemic where it was expected that children’s well-being would be negatively affected. Our planned next stage of research is to consolidate these findings by conducting an evaluation of the CP3 using a stepped-wedge cluster randomized controlled study.

### Co-design With Children and Their Communities at the Heart of CP3

The qualitative findings demonstrated the positive flow on the effect of children making active decisions about the service where they play, learn, and grow. Research highlights that central to achieving a shift in OSHC service delivery is the need to listen to children’s voices [25,26]. As highlighted by Flückiger et al [27] and echoed in Australian research [28], educators need to be able to listen to children to develop policies and practices that directly respond to their needs and perspectives. This process of enabling OSHC services and their educators to listen and respond to children’s voices is structurally supported through the CP3 as a best-practice programming tool.

The wider OSHC community (including educators and CP3 volunteer mentors) also described benefiting from the CP3 co-design process. Research highlights that, for educators, having a voice in service delivery is critical in addressing workforce issues that have arisen in recent years [29]. The educator voice is recognized in the Australian National Quality Standard [7], which emphasizes the need for democratic practices and collaborative decision-making across all aspects of service delivery. The qualitative results from this process evaluation indicate a positive impact of the CP3 on educator well-being and sense of community. This warrants further quantitative research to understand whether the CP3 providing a framework for such collaborative decision-making practices influences educators’ workplace well-being.

### Creative Engagement

Past Australian qualitative research has highlighted that, when asked, children emphasize the importance of friendship, play, and choice of specific activities in OSHC [28]. All these ideas are echoed in the CP3 principles of Broaden Horizons, Inspire and Engage, and Connect Communities. Creative engagement through these channels is a way of directly and indirectly enhancing child social, emotional, physical, and cognitive well-being. The qualitative findings of this study highlighted that more susceptible children were more likely to engage positively with CP3 activities as compared with regular OSHC. There was a distinct change in how they engaged socially and emotionally, forming positive connections through play via the CP3 activities they had themselves co-designed. This high-quality programming process through the CP3 is important as there is research evidence suggesting that, in other childcare settings, those that are most likely to benefit from high-quality programming are children who experience circumstances of disadvantage [30].

### The Reality of CP3

The main criticism of the CP3 relates to its potential to add to the workload of OSHC service providers if not properly resourced. For example, the level of paperwork was a concern raised in our formative and process evaluation findings. Although this may have been because educators had conflated research-related paperwork with the CP3 itself, this aspect remains important. Research in the early childhood education sector has highlighted that issues such as paperwork are a major impediment to workplace well-being and the educational effectiveness of educators [31], which can, in turn, influence the quality of OSHC service provision and child outcomes [32,33]. The digitization of CP3 implementation and support tools is planned to minimize the administrative burden of the CP3. This is also intended to augment the CP3’s reach to OSHC services located in regional and rural areas.

In line with the “Shaping our Future” strategy, which focuses on supporting Australia’s childcare workforce over the next decade [34], if the CP3 is to be successful in the future, it needs to positively affect educator well-being and not be a source of burden. Thus, the CP3 coordinator and CP3 training infrastructure being in place to support OSHC services to deliver, fully resource, and upskill the workforce will be essential to the ongoing success of the CP3. Workforce issues are currently of major concern across the childcare sector, and these types of well-being–focused programs can only be implemented and provide value if funding and resources are sufficient to ensure effective implementation.

Another concern was that the quality of the program could fluctuate depending on the CP3 champion at the OSHC service. This could be attributed to some of the aforementioned workforce issues. However, this may also be because implementation practices differed between services. To understand and be able to support services with this variability, a CP3 fidelity tool is being developed. It is envisaged that a CP3 fidelity tool will (1) provide a structured framework for quality assurance and quality improvement of CP3 delivery; (2) allow services to identify and address any implementation or resourcing issues early; and (3) provide a means for researchers to identify components of the CP3 model, if any, that are critical for positive outcomes.

### Strengths and Limitations of the Research

For the first formative evaluation, the CP3 was piloted at 1 OSHC site only, which meant that the sample numbers for the child and educator surveys were very small and may be biased toward the specific sociocultural-economic aspects of that area. The CP3 was subsequently rolled out across an additional 5 sites for the process evaluation so as to gather further evidence in more diverse settings and with a larger sample size. In this evaluation phase, mixed methods data were gathered from children, but only qualitative data could be gathered from educators given funding constraints and the fact that the primary target of the intervention were children. For the process evaluation preliminary effectiveness, only 58 children could be matched across the 2 time points for the SDQ surveys. This was due to children at each time point either joining or leaving the service or the service not completing the routine data outcome monitoring at the time because of unforeseeable circumstances (eg, fires, floods, and COVID-19–related lockdowns). The average attrition rate across all sites from baseline to follow-up was 33.8% (88/122), which increased to 52.5% (58/122) in the matched sample. This attrition is high and poses a threat to the validity of the findings; thus, statistical testing must be interpreted with caution. A real-world randomized cluster stepped-wedge trial is being carried out at regional and urban OSHC sites in NSW, Australia, and will provide more comprehensive evidence for the CP3 model from both a child and an educator perspective.

A critical strength of the CP3 model is that it has undergone iterative development following recommendations of the Medical Research Council guidelines for developing complex interventions [14]. After the initial co-design of the CP3 model [6], the use of a 2-step formative and process evaluation enabled further program design to be agile and actively respond to the identified needs as they arose, for example, the development of a fidelity measure and additional resources to support educators in explaining the CP3 to the community. By using this approach, the CP3 model can grow and be improved upon in real time as a program and service improvement process.

### Conclusions

There is strong academic evidence for the developmental effectiveness of providing high-quality programming (intellectually stimulating, emotionally supportive, and providing socially engaging learning experiences) in early childcare settings [29]. This should be extended to OSHC in the primary school years. Providing high-quality OSHC programming is an investment in children’s futures given that OSHC is the fastest-growing childhood education and care sector in Australia [5]. OSHC sites need to extend beyond simply existing as “convenient care” and be valued as a place where children’s well-being is supported [5]. The early evidence for the CP3 is promising and may provide a unique opportunity to listen and respond to the voices of children in OSHC and those that support them. To ensure future sustainability and scalability, there must be sufficient resources to ensure that such programs do not burden an already overstretched workforce. The CP3 provides OSHC with a much-needed framework and high-quality program planning tool, which promotes the development of tailored interventions depending on the unique needs and preferences of those who will use them.

## Appendix

Multimedia Appendix 1 Child and adult formative evaluation demographics.

Multimedia Appendix 2 Staff (n=5) and volunteer (n=2) satisfaction with Connect, Promote, and Protect Program items during term 1 in 2020.

Multimedia Appendix 3 Number of completed Strengths and Difficulties Questionnaires at baseline and follow-up by outside school hours care site.

Multimedia Appendix 4 Educator baseline and follow-up results for each Strengths and Difficulties Questionnaire (SDQ) scale by 4-fold SDQ categories.

## Abbreviations

ACECQA: Australian Children’s Education and Care Quality Authority
CP3: Connect, Promote, and Protect Program
MD: mean difference
NSW: New South Wales
OSHC: outside school hours care
SDQ: Strengths and Difficulties Questionnaire
