# Supplementary material for: Supporting Children’s Social Connection and Well-Being in School-Age Care: Mixed Methods Evaluation of the Connect, Promote, and Protect Program
Source: JMIR Pediatr Parent. 2023 Jul 25;6:e44928. doi: 10.2196/44928 (PMC10410534; doi:10.2196/44928)
Supplement: Multimedia Appendix 1 [file pediatrics_v6i1e44928_app1.docx]

***Multimedia Appendix 1.*** Child and adult formative evaluation demographics

| **Demographics** | **Child** | | **Adult** | |
| --- | --- | --- | --- | --- |
|  | **n** | **(%)** | **n** | **(%)** |
| Time point |  |  |  |  |
| *During Term 3 2019* | 11 | 37.9 | 5 | 15.6 |
| *End of Term 4 2019* | 10 | 34.5 | 9 | 28.1 |
| *End of Term 1 2020** | 8 | 27.6 | 18 | 56.3 |
| Gender |  |  |  |  |
| *Girl/Female* | 17 | 60.7 | 13 | 13.3 |
| *Boy/Male* | 11 | 39.3 | 2 | 86.7 |
| Year at school |  |  |  |  |
| *Kindergarten* | 3 | 11.1 | - | - |
| *1* | 0 | 0.0 | - | - |
| *2* | 6 | 22.2 | - | - |
| *3* | 5 | 18.5 | - | - |
| *4* | 6 | 22.2 | - | - |
| *5* | 6 | 22.2 | - | - |
| *6* | 1 | 3.7 | - | - |
| Language at home |  |  |  |  |
| *English* | 24 | 85.7 | - | - |
| *Other* | 4 | 14.2 | - | - |
| Role |  |  |  |  |
| *OSHC educators* | - | - | 10 | 31.3 |
| *Volunteer* | - | - | 6 | 18.8 |
| *Parent or guardian* | - | - | 9 | 28.1 |
| *Other* | - | - | 7 | 21.9 |
| Age |  |  |  |  |
| *18-24 years* |  |  | 3 | 20.0 |
| *25-34 years* |  |  | 2 | 13.3 |
| *35-44 years* |  |  | 6 | 40.0 |
| *45-54 years* |  |  | 2 | 13.3 |
| *65+ years* |  |  | 2 | 13.3 |

** time point 3 data collection was impacted by the COVID-19 lockdown*
