# Supplementary material for: Supporting Children’s Social Connection and Well-Being in School-Age Care: Mixed Methods Evaluation of the Connect, Promote, and Protect Program
Source: JMIR Pediatr Parent. 2023 Jul 25;6:e44928. doi: 10.2196/44928 (PMC10410534; doi:10.2196/44928)
Supplement: Multimedia Appendix 2 [file pediatrics_v6i1e44928_app2.docx]

***Multimedia Appendix 2.*** Staff (n=5) and volunteer (n=2) satisfaction with CP3 items during Term 1 2020

|  |  |
| --- | --- |
