# Supplementary material for: Supporting Children’s Social Connection and Well-Being in School-Age Care: Mixed Methods Evaluation of the Connect, Promote, and Protect Program
Source: JMIR Pediatr Parent. 2023 Jul 25;6:e44928. doi: 10.2196/44928 (PMC10410534; doi:10.2196/44928)
Supplement: Multimedia Appendix 3 [file pediatrics_v6i1e44928_app3.docx]

***Multimedia Appendix 3.*** Number (n) of completed SDQs at Baseline and Follow-up by OSHC site

|  | Number (n) of SDQ responses | | |
| --- | --- | --- | --- |
| OSHC | Baseline  (Educator) | Follow up  (Educator) | Educator SDQ completion rate |
| Site 1 (CH) | 0 | 0 | NA |
| Site 2 (MLC) | 42 | 35 | 83.3% |
| Site 3 (M) | 29 | 29 | 100% |
| Site 4 (NP) | 19 | 17 | 89.5% |
| Site 5 (E) | 32 | 7 | 21.9% |
| Total | 122 | 88 | 66.2% |
