# Supplementary material for: Supporting Children’s Social Connection and Well-Being in School-Age Care: Mixed Methods Evaluation of the Connect, Promote, and Protect Program
Source: JMIR Pediatr Parent. 2023 Jul 25;6:e44928. doi: 10.2196/44928 (PMC10410534; doi:10.2196/44928)
Supplement: Multimedia Appendix 4 [file pediatrics_v6i1e44928_app4.docx]

**Multimedia Appendix 4.** Educator baseline and follow-up results for each SDQ scale by four-fold SDQ categories

|  | | Baseline | | Follow up | |
| --- | --- | --- | --- | --- | --- |
|  | | Number (n) | Percent | Number (n) | Percent |
| Emotional Problems Score | Close to Average | 98 | 73.7% | 65 | 74.7% |
|  | Slightly raised | 10 | 7.5% | 4 | 4.6% |
|  | High | 8 | 6.0% | 7 | 8.0% |
|  | Very high | 17 | 12.8% | 11 | 12.6% |
|  | Subtotal | 133 | 100.0% | 87 | 100.0% |
| Hyperactivity Problems Score | Close to Average | 95 | 70.9% | 77 | 87.5% |
|  | Slightly raised | 19 | 14.2% | 6 | 6.8% |
|  | High | 9 | 6.7% | 2 | 2.3% |
|  | Very high | 11 | 8.2% | 3 | 3.4% |
|  | Subtotal | 134 | 100.0% | 88 | 100.0% |
| Peer Problems Score | Close to Average | 81 | 60.4% | 54 | 61.4% |
|  | Slightly raised | 25 | 18.7% | 17 | 19.3% |
|  | High | 11 | 8.2% | 10 | 11.4% |
|  | Very high | 17 | 12.7% | 7 | 8.0% |
|  | Subtotal | 134 | 100.0% | 88 | 100.0% |
| Prosocial Behaviours Score | Close to Average | 74 | 55.6% | 63 | 71.6% |
|  | Slightly lowered | 17 | 12.8% | 7 | 8.0% |
|  | Low | 14 | 10.5% | 2 | 2.3% |
|  | Very low | 28 | 21.1% | 16 | 18.2% |
|  | Close to Average | 74 | 55.6% | 63 | 71.6% |
| Conduct Problems Score | Close to Average | 89 | 66.4% | 46 | 52.3% |
|  | Slightly raised | 13 | 9.7% | 9 | 10.2% |
|  | High | 10 | 7.5% | 10 | 11.4% |
|  | Very high | 22 | 16.4% | 23 | 26.1% |
|  | Subtotal | 134 | 100.0% | 88 | 100.0% |
| Impact Score | Not at all (no impact) | 40 | 60.6% | 59 | 89.4% |
|  | Only a little | 10 | 15.2% | 0 | 0.0% |
|  | A medium amount | 1 | 1.5% | 1 | 1.5% |
|  | A great deal | 15 | 22.7% | 6 | 9.1% |
|  | Subtotal | 66 | 100.0% | 66 | 100.0% |
